# Systemic solutions to emergency department boarding: the hospitalist's perspective on the need for broader alignment and collaboration

**DOI:** 10.1093/haschl/qxaf168

**Published:** 2025-08-26

**Authors:** Dahlia Rizk, Joshua Lapps, Jennifer B Cowart

**Affiliations:** Division of Hospital Medicine, Mount Sinai Health System, Icahn School of Medicine, New York, NY 10029, Public Policy Committee, Society of Hospital Medicine, Philadelphia, PA 19130, USA; Society of Hospital Medicine, Philadelphia, PA 19130, USA; Division of Hospital Internal Medicine, Mayo Clinic Jacksonville,Florida 32224, Public Policy Committee, Society of Hospital Medicine, Philadelphia, PA 19130, USA

**Keywords:** ED boarding, healthcare systems, hospitalists, throughput, quality of care, access

## Abstract

Boarding of admitted patients in the emergency department (ED) is increasingly common and frequently discussed as an issue related to ED throughput. However, ED boarding should instead be seen as a symptom of systemic issues relating to hospital capacity and throughput, requiring a multidisciplinary team approach locally, as well as health system-level policy intervention. Hospitalist clinicians frequently care for boarding patients and are experts in clinical care of acutely ill patients and with experience are often experts in quality improvement as well. In this commentary, we call upon hospitalists and emergency medicine (EM) physicians to tackle this problem together, along with healthcare administrators, to look for potential solutions from the local hospital level up to US federal healthcare policy.

## Introduction

Patients who are admitted from the emergency department (ED) but are unable to move to a hospital room in a certain timeframe are considered to be “boarding.” Patients with prolonged ED boarding stays are at risk for fragmented care, delayed procedures and interventions, and potential clinical deterioration, which lead to increased length of stay, morbidity, and mortality. Boarding is often considered an ED-related problem, but instead should be viewed as a symptom of systemic issues.

Hospitalists are experts in acute illness management and often care for boarding patients. The Society of Hospital Medicine (SHM) considers quality improvement, equitable resource allocation, and multidisciplinary care to be core competencies for hospitalists.^1^ In mature hospital medicine divisions and healthcare systems, hospitalists have evolved to lead committees on readmissions, patient safety, throughput, and utilization management. The systemic problems leading to ED boarding, from local hospital challenges, workforce shortages, and misaligned reimbursement and technical policies, require collaboration to overcome. In this commentary, we call upon hospitalists and emergency medicine (EM) physicians to tackle this challenge together, sharing insights from our perspective as hospitalists.

## ED boarding and hospital occupancy are inextricably linked

Emergency department boarding has increased in frequency and national attention while hospital occupancy rates are also climbing. During the COVID-19 pandemic, mean national hospital occupancy jumped nearly 11 points to 75.3%, while staffed hospital beds declined by 16%.^2^ One study showed that hospital occupancy above 85% correlated with increased boarding beyond 4 h.^3^ However, ED performance metrics, such as left without being seen and time to admission decision, are not typically adjusted for hospital census or other exogenous factors. Prolonged boarding has been shown to be predictive of lower patient satisfaction with both the ED and the overall hospitalization^4^ as well as increasing morbidity and mortality in older patients.^5^

Boarding is a major concern for hospitalists. The Society of Hospital Medicine's 2025 State of Hospital Medicine Report asked about strategies hospitalist groups use to address or manage ED boarding in their institutions.^6^ Table 1 shows self-reported approaches from a representative national sample of 224 adult hospital medicine groups.

**Table 1.** Strategies adult hospital medicine groups employ to address challenges with ED holds/boarding^a^.

Avoidable admissions: discharge from ED and establish care with rapid follow-up clinics	69.2%
Discharge lounge	45.1%
Hallway boarding: moving patients to the hallway on floors vs holding in ED	42.9%
Partnership with postacute services	41.1%
Designated hospitalist teams for ED holds	29.0%
Hospital at home	27.7%
ED discharge/remote patient monitoring	12.9%
Other	9.4%

Data excerpted from the 2025 State of Hospital Medicine Report published by the Society of Hospital Medicine.

^a^Groups were instructed to select all that apply, so percentages will not add up to 100.

## Local challenges and collaborative solutions

While each hospital or local health system has unique challenges, common themes emerge as opportunities to collaborate.

### ED disposition

Assigning ED patients to the correct level of care and site of service requires staffing resources, including case management, physical therapy services, and social work. Service availability, such as outpatient care access, ED diagnostic testing, and specialist follow-up, can be a significant barrier to patient throughput. Sufficient access to these services can help with triage and, in some cases, diverting patients to outpatient management instead of a hospital admission. Communication between EM and hospitalist teams is paramount to ensure efficient patient transitions of care and to identify which factors warrant escalation to leadership.

### Upon admission

Hospitalized patients are getting sicker: the American Hospital Association reported that from 2019 to 2024, the acuity of hospitalized patients increased by 3%.^7^ Increased acuity means patients have more comorbidities, higher severity of illness, and increased complexity, requiring more clinician time. If timely access to inpatient teams is a significant factor for ED boarding in their hospital, hospitalists and EM physicians should jointly bring this to leadership attention. Investment in other tools may be useful to add inpatient capacity, such as hospital at home, which has shown promise in improving mortality and reducing costs for appropriate patients.^8^

### Throughout inpatient stay

Staffing is a major barrier in hospital capacity, with overall US staffed hospital beds declining from 802 000 pre-COVID-19 pandemic to 674 000 postpandemic.^2^ Beds may be physically available but cannot be used without appropriate personnel including physicians, nursing, support staff, and environmental services. Data-driven staffing management may improve patient flow during their hospitalization, but requires administrative buy-in and prioritization. Capacity command centers may enable systems to manage patient flow and load leveling across a system's entire set of available resources, although more research is necessary to understand their full impact on patient outcomes.

Specialty and acute patient service availability also impedes throughput. Weekend coverage is often lighter for medical and procedural specialties, as well as critical ancillary services. Hospital leadership can facilitate throughput and acute patient care by ensuring 7-day access to consultants, nutritionists, imaging, procedures, and disposition planning services such as case management and physical/occupational therapy. A 2015 study suggested that interventions to improve weekend care positively affected throughput and decreased length of stay.^9^ To ensure returns on these investments, staffing can target the service lines with greatest need for the highest impact. Administrative data on provider specific metrics and turnaround time for procedures can aid hospitals in reducing variability and improving use of evidence-based best practices in all hospital-based teams. Physician leaders in each of these spaces should have access to data to benchmark and improve processes in their domains.

### Prior authorization delays

Payer barriers, such as prior authorizations for medications or postacute care services, commonly keep patients in the hospital longer than necessary. Hospitals should track avoidable days related to payer issues and manage contracts accordingly. Mandated public transparency and accountability around payer-related delays leading to excess days and denials may also help streamline patient access and care.

### Discharge

Hospitalists commonly face challenges such as limited postacute bed availability, prior authorizations for rehabilitation services, and other administrative issues that contribute to delays in discharge. Home health services may have constrained availability due to declining reimbursement.^10^ These impediments can be exacerbated for vulnerable patients, including those with substance use disorders, those who lack consistent housing, and populations who are un- or underinsured. Variability in coverage for postacute care across different payers and states can make it difficult to create consistent hospital processes to maximize efficient patient throughput. Navigating complex discharge situations such as patient and family disagreements with care plans, or lack of family support necessitates investments in case management and connections with community resources.

## Systemic and policy solutions

Hospitalists and EM physicians also have an opportunity to collaborate on national policy to address system-level factors contributing to ED boarding. There are numerous policy fixes that each address a small piece of the puzzle, but together can cumulatively impact the quality and efficiency of care for patients in EDs and hospitals nationwide. Table 2 contains potential policy approaches that can improve hospital throughput and directly or indirectly reduce ED boarding. Some policies, particularly in the Medicare program, were created decades ago and should now be reevaluated holistically in light of changes to clinical care, length of stay, and acuity of hospital patients. In recent months, some of the policy priorities detailed below have seen movement, including Centers for Medicare & Medicaid Services proposals for maintaining expanded telehealth and payer commitments to reforming prior authorization. Of concern, upcoming changes to Medicaid coverage could exacerbate care access and ED boarding. The cumulative impact of these changes requires study as they are implemented.

**Table 2.** Potential policy and local solutions for collaborative advocacy.

Issue	Potential federal policy solutions	Potential local solutions
Inadequate access to postacute care, including skilled nursing facilities (SNFs) and home care	Eliminate requirement that Medicare patients have 3 inpatient days for SNF coverage Support policies and payment models to increase access to home care	Optimizing regional capacity for postacute care including developing relationships between hospitals and SNFs Refining appropriateness of referrals to postacute facilities and home care by increasing mobility in the hospital
Underutilized telemedicine programs to address outpatient and regional care	Maintain and expand telehealth flexibilities (proposed in 2026 Physician Fee Schedule proposed rule)	Invest in and grow local telemedicine programs to grow system capacity. This includes ensuring buy-in from consultants in system and region to participate
Stability of the physician workforce	Support and expand high-skilled immigration pathways to address immediate clinician shortages, particularly in rural and underserved areas, including reauthorizing/expanding the Conrad State 30 program Minimizing administrative burdens, including mandated paperwork requirements and improving use of technology for documentation reduction. Continued renewal of the Lorna Breen Act and other focused mental health legislation	Ensuring systems have support for the hiring, on-boarding, and international medical graduates. Optimizing use of all-levels of clinical staff support, including integrating nurse practitioners and physician assistants/associates Identifying and streamlining burdensome workflows in electronic health record (EHR) and other systems Eliminate barriers to access to mental health resources and support wellbeing initiatives
Misalignment of performance measures	Advocate for Medicare and other payers to align quality measures between ED and hospital settings	Work with hospital administration to identify areas of misaligned measures or incentives used at the local level and develop alternatives
Physical limitations to capacity in the hospital	Maintain and expand the Hospital at Home waiver and similar programs as capacity building tools	Create and support hospital at home pilots and programs at your hospital/system
Payer prior authorizations and other administrative barriers	Monitor new Medicare policies around prior authorization and payer commitments to reducing burden. Work toward additional policy solutions as needed	Identify the largest administrative barriers to patient throughput and partner across service lines (potentially across systems) to leverage data for advocacy back to health plans

## Conclusion

Emergency department boarding is a multifaceted issue that requires a collaborative approach among ED physicians, hospitalists, hospital administrators, and policymakers. By focusing on communication, case management, and administrative strategies to improve patient flow, hospitals can mitigate the negative impacts of boarding on patient care. By addressing systemic barriers to discharge and advocating for policy changes that improve access to care, hospitalists and ED physicians can work together to create a more efficient and patient-centered healthcare system. The time to act is now: by uniting hospital teams and partnering with leadership as well as advocating for policy change, we can build a healthcare system that meets the needs of patients *and* clinicians.

## Supplementary Material

qxaf168_Supplementary_Data
